# Generation of Ugt1-Deficient Murine Liver Cell Lines Using TALEN Technology

**DOI:** 10.1371/journal.pone.0104816

**Published:** 2014-08-13

**Authors:** Fabiola Porro, Luka Bockor, Alessia De Caneva, Giulia Bortolussi, Andrés F. Muro

**Affiliations:** International Centre for Genetic Engineering and Biotechnology (ICGEB), Trieste, TS, Italy; University of Nantes, France

## Abstract

The Crigler-Najjar Syndrome Type I (CNSI) is a rare genetic disorder caused by mutations in the Ugt1a1 gene. It is characterized by unconjugated hyperbilirubinemia that may result in severe neurologic damage and death if untreated. To date, liver transplantation is the only curative treatment. With the aim of generating mutant cell lines of the Ugt1 gene, we utilized the TALEN technology to introduce site-specific mutations in Ugt1 exon 4. We report a fast and efficient method to perform gene knockout in tissue culture cells, based on the use of TALEN pairs targeting restriction enzyme (RE) sites in the region of interest. This strategy overcame the presence of allele-specific single nucleotide polymorphisms (SNPs) and pseudogenes, conditions that limit INDELs' detection by Surveyor. We obtained liver-derived murine N-Muli cell clones having INDELs with efficiency close to 40%, depending on the TALEN pair and RE target site. Sequencing of the target locus and WB analysis of the isolated cell clones showed a high proportion of biallelic mutations in cells treated with the most efficient TALEN pair. Ugt glucuronidation activity was reduced basal levels in the biallelic mutant clones. These mutant liver-derived cell lines could be a very useful tool to study biochemical aspects of Ugt1 enzyme activity in a more natural context, such as substrate specificity, requirement of specific co-factors, the study of inhibitors and other pharmacological aspects, and to correlate enzyme activity to the presence of specific mutations in the gene, by adding back to the mutant cell clones specific variants of the Ugt1 gene. In addition, since genome editing has recently emerged as a potential therapeutic approach to cure genetic diseases, the definition of the most efficient TALEN pair could be an important step towards setting up a platform to perform genome editing in CNSI.

## Introduction

UDP-glucuronosyl transferases (UGTs) catalyze glucuronidation of a great variety of compounds. They are classified into two subfamilies, UGT1 and UGT2 [1]. Each subfamily is composed of several different isoforms, expressed in a tissue-specific manner and having substrate specificity [2]. The most important member of the UGT1 subfamily is UGT1a1, which is mainly expressed in the liver, and is the only enzyme able to conjugate bilirubin. Deficiency of this isoform results in Crigler-Najjar Syndrome Type I (CNSI), a rare genetic disorder characterized by unconjugated hyperbilirubinemia [3], [4]. Persistent unconjugated hyperbilirubinemia may cause severe neurologic damage and result in death by kernicterus if untreated. To date, liver transplantation is the only curative treatment.

The study of the mechanisms of disease and the effects of pharmacological therapies rely in the use of specific cellular and animal models containing mutant versions or variants of the causative gene. The absence of rapid and efficient tools to modify the genome limited the availability of cellular models. However, in the last few years the development of a new generation of nucleases having a very high sequence-specificity allowed the rapid and efficient modification of the loci of interest [5]–[8]. These nucleases are known as Zinc Finger nucleases (ZFN), Transcription Activator Like Effector Nucleases (TALEN) and Clustered Regularly Interspaced Short Palindromic Repeats (CRISPR) together with the CRISPR associated nuclease 9 (Cas9), also denominated RNA Guided Endonucleases (RGEN). They generate a precise single- or double- strand break at a specific locus [6], [9]. Among the mentioned nucleases, TALENs integrate two important features: the ease of construction that ZFN lack, and higher specificity than RGENs have produced so far. They consist of two domains: a customizable DNA binding domain and the catalytic domain of FokI endonuclease [7], [9]. TALENs work as heterodimers in which each monomer binds 15–20 bp of DNA that flanks a 15–24 bp spacer region. Once both monomers bind to the target sequence, the FokI endonuclease domains introduce a double strand break (DSB). When homologous donor DNA is present, DSBs are repaired by Homology-directed repair (HDR), a pathway that corrects the damage and is used for “gene editing”. Alternatively, when no donor DNA is present, the damage is repaired by non-homologous end-joining (NHEJ), which results in insertions or deletions (INDELs) in the target site [6], [7]. Consequently, when the target site is in the protein coding sequence, two-thirds of the repairs will cause a frameshift, which may lead to translational termination and loss of function [6], [7].

In the present work, we used the TALEN technology to introduce mutations in the Ugt1 gene of mouse liver-derived N-Muli cells. Our strategy was based in the inactivation of the gene by targeting restriction enzyme sites present in the exon 4 of the Ugt1. We successfully generated mouse liver cells containing mono- and bi-allelic mutations in the exon 4 of the Ugt1 gene.

## Materials and Methods

### Generation of TALENs pairs

To design the different TALEN pairs we used the online TAL Effector Nucleotide Targeter (TALENT) software [10]. To generate the TALEN plasmids we used the “Golden Gate TALEN and TAL effector Kit 2.0 (Addgene, cat. #1000000024), as previously described [11]. The generated TALENs are based on the GoldyTALEN scaffold, that contains a deletion of 152 amino acids (aa) at the N-terminal domain and a 63 aa C-terminal residue [12]. This scaffold was previously shown to be the most efficient one [9]. The target sequences of the TALENs are indicated in Figure S3.

### Surveyor assay

To set up Surveyor Assay genomic DNA (gDNA) was extracted from wild type (WT) and UGT1A1 KO animals by overnight proteinase K digestion of tail biopsies. Plasmids containing WT and mutant (single point mutation) sequence were used as control. Several primer pairs were tested for both gDNA and plasmid DNA, with the highest yield and a single PCR product amplified with the LSur1F and LSur1R primer pair. For the Surveyor Assay the DNA was then PCR-amplified with LSur1F and LSur1R primers pair (see Table S1) under the following conditions: 95°C 5 min, then 94°C 30 s, 70°C 30 s, 72°C 30 s for 35 cycles, with 3 min of final extension step at 72°C. After PCR the amplicons were treated with Surveyor (Surveyor mutation detection kit, Transgenomic) as per manufacturer's instructions. Products were resolved by agarose gel electrophoresis (2% agarose gel).

### Generation of the luciferase reporter vector

The pGL3-Linker vector was obtained from the pGL3-control vector by inserting the 582 bp long NcoI-BclI fragment (purified from the pPGL-3 control vector) and a 31 bp linker (containing the BclI-EcoRI-EcoRV-BamHI-PstI-NarI restriction enzyme sites) into a pGL-3 vector (having the BamHI site inactivated) previously cut with NcoI and NarI restriction enzymes. The final construct was verified by restriction enzyme digestion and sequencing.

To generate the pGL3-mUGT1a1 WT vector, the 706 bp long BamHI-BamHI fragment of the mUgt1a1-Ex4 WT, (previously cloned and sequenced in our lab), was cloned into the BamHI site of the pGL3-linker plasmid.

### Transient cell transfections and dual luciferase assay

24 h before transfection HEK293 cells were plated in 24 wells/plate in complete DMEM (10%FBS and antibiotic). At the day of transfection the cells were at 50–70% of confluence. The cells were co-transfected with 40 ng of Renilla (phRG-TK, Clontech), 1.6 µg of pGL3-Linker (as negative control) or pGL3-mUGT1a1-ex4 plasmids and increasing amounts of the TALEN plasmids pair (400 ng, 800 ng and 1.6 µg) using Lipofectamine 2000 as described by the manufacturer (InVitrogen). The day after, 500 µl of complete DMEM (containing 20% FBS) were added. 72 h after transfection cells were harvested and TALEN cutting efficiency was indirectly determined by quantifying luciferase activity with the Dual Luciferase assay (Promega). Briefly, cells were washed and lysed with 100 µl of PLB 1× (Passive Lysis Buffer -PROMEGA), incubated 15 min at RT and centrifuged at top speed for 1 min. Firefly Luciferase activity was first measured by adding 30 µl of LAR II (Luciferase Assay Reagent II) to 30 ul cell lysate; then 30 µl of Renilla luciferase substrate (Stop & GLO) were added and renilla luciferase activity was determined with a single-sample 20/20^n^ luminometer from Turner Biosystem.

### Generation of mutant stable cell clones by TALEN transfection

N-Muli cells (ATCC CRL-1638) were cultured in D-MEM with 10% FCS. Due to the low transfection efficiency of N-Muli cells (15–20%), we performed two successive rounds of transfection: a) 2×10^6^ N-MuLi cells were transfected with 2 µg of p-EGFP-C2-plasmid (for G418 selection, Gentech) and 3 µg of both TALEN right and left arms cloned in the pC-Goldy plasmid (Mnl, Nco, Nla or BstXI), by using the Effectene reagent (Qiagen) and following the manufacturer's instructions. Cells were cultured at 37°C for 24 h (with 10% FBS) and 72 h later G418 (250 µg/µl) was added; b) When cells reached 70–80% of confluence they were transfected as indicated above, but with 2 µg of pMCS-AAT-PB:PGKpuro-deltaK (Welcome Trust Sanger Institute) for puromycin selection, instead of the p-GFP-plasmid. 72 h after transfection Puromycin at 2 µg/µl was added and single resistant clones were selected, picked and individually cultured.

### Genomic DNA preparation, PCR of genomic DNA, digestion with restriction enzymes to analyze INDELs, and DNA sequencing

Genomic DNA preparation was performed as previously described [13]. Briefly, cells were lysed in 50 mM TRIS pH 7.5, 100 mM EDTA, 0.5%SDS and 200 µg/ml of protease K for 1 hour at 37°, centrifuged for 10 min at maximum speed, genomic DNA precipitated with 2-propanol and resuspended in TE after centrifugation and washing.

Genomic PCR was performed from 10–30 ng of DNA, by using the primers indicated in Table S1. The PCR product was then digested with the restriction enzymes specific for each TALEN pair (NlaI, Nco, BstXI and MnlI), and the RE-resistant fraction was purified, cloned into pUC19 and sequenced.

### Western blot analysis of positives clones

Protein cell extracts were prepared as already described [13]. Briefly, cells were lysed in 50 mM Tris pH7.5, 150 mM NaCl, 1% NP40 (containing protease inhibitors, Complete-Roche), centrifuged to clarify debris, and protein concentration was quantified by Bradford (Biorad). The same amounts of protein extracts (100 µg for cell clones, and 30 for liver) were loaded on 10% SDS-PAGE, blotted onto nitrocellulose, incubated with the primary antibody anti-mouse UGT1a1 (Santa Cruz, cat# sc-25847, 1∶400), incubated with a secondary antibody (anti-rabbit HRP-conjugated - DAKO) and developed by ECL (Pierce) as described [13].

### Ugt enzyme activity assay

The UGT1a1 activity was determined by UGT-GloTM assay kit in the microsomal samples according to manufacturer's instructions (Promega, Madison, WI). The rationale of the assay resides in the use of a UGT Multienzyme Substrate, which reacts with the luciferin detection reagent to give light that can be quantitated by a luminometer. When the UGT substrate is glucuronidated it will no longer react with the luciferin detection reagent to give light. To determine enzyme activity, two reactions are set up per sample, one with the co-substrate UDPGA and the other without. Only the reaction with the UDPGA will produce glucuronidated substrate if UGTs are present and active. Total UGT activity is then quantified by the difference in emitted light between the two reactions. To determine UGT1 activity, we prepared microsomes from wt N-Muli, Mnl20, Nco20 and Nco25 cells. We used liver microsomes prepared from Ugt1 KO mice and WT mice as controls [13], in addition to the microsomal preparations (positive control) of the UGT-GloTM assay kit (Promega Madison, WI). Microsomes (10 µg from cell clones, 1 µg from mouse liver and 4 µg from positive control) were incubated with 16 mM UDPGA, 20 µM multi enzyme substrate and 0.35 mg/ml digitonin (Sigma) at 37°C for 150 min. After which 40 µl of reconstituted luciferin detection reagent containing D-Cysteine was added and the luminescent signal was allowed to stabilize for 20 min at room temperature. Luminescence was read using a multiplate reader (Perkin Elmer Envision Plate Reader, Walthan, MA, USA). Data was expressed as percentage of the luminescent substrate consumed.

## Results

### Targeting restriction enzyme sites with TALENs to avoid interference of strain-specific *Ugt1* SNPs and/or pseudogenes

In order to determine TALEN efficacy in targeting the mUgt1 gene, we initially performed the Surveyor assay using PCR amplification products obtained from plasmids containing the wt or mutated sequences of the Ugt1 gene exon 4 and flanking introns. These DNA fragments, originating from 129Sv/J ES cells, had been previously cloned and mutated in our laboratory, and used to generate a mouse strain containing one base deletion in the exon 4 of the Ugt1 gene [13]. To our expectations, the Surveyor assay resulted in two bands of the predicted size when wt and mutant PCR products were mixed in the presence of Surveyor (192 and 324 bp, Figure S1, lanes 2–4). Surprisingly, when we performed the Surveyor assay using the PCR products amplified from wt and mutant Ugt1 mice genomic DNA, we obtained a laddering pattern of bands, regardless of the source of DNA used for the reaction, indicating the presence of heteroduplexes (Figure S1, lanes 5–7). A similar result was obtained when we tested genomic DNA from N-Muli hepatoma cells (data not shown), which originates from the C57Bl/6 mouse strain.

Since mutant mice had been backcrossed with C57Bl/6 mice for 9 generations to obtain a homogeneous genetic background, but the mutation was generated in 129Sv/J ES cells, we suspected the presence of strain-specific variants and/or pseudogenes that might produce mismatches and unexpected bands in the Surveyor reaction. To verify this possibility, we cloned the PCR product of wt 129Sv/J genomic DNA and sequenced nine (9) independent clones. As reported in Figure S2, we compared those clones with the available C57Bl/6 GenBank reference sequence and we observed the presence of mismatches among some of the 129Sv/J clones with the reference C57Bl/6 sequence and, unexpectedly, among the 129Sv/J-derived clones themselves. The sequences were categorized into two distinct groups according to the mismatches, as indicated in Figure S2. We speculate that the failure in the Surveyor assay could be due to the presence of strain-specific polymorphisms in the Ugt1 exon 4, and/or to a Ugt1a1 pseudogene. The presence of a pseudogene of the Ugt1 gene was previously suggested by data presented by Miles [14], Seldin [15], [16], Lathman et al [17], MGI and NCBI (UDP glucuronosyltransferase 1 family, polypeptide A1, related sequence 1, Ugt1a1-rs1, Jackson Lab, http://www.informatics.jax.org/marker/MGI:98899; NCBI, http://www.ncbi.nlm.nih.gov/gene/111282). However, no active link to the pseudogene sequence was yet available in the mentioned gene banks and we failed to identify the Ugt1a1-rs1 pseudogene in the currently available gene banks (not shown).

**Figure 1:**
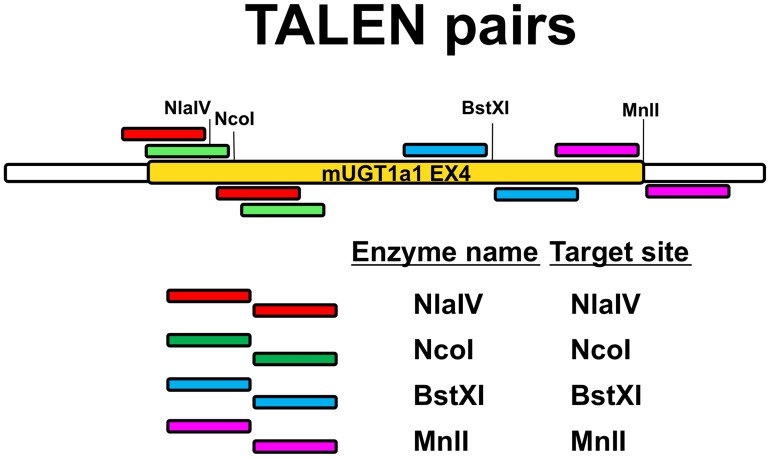
Scheme of the WT exon 4 showing the position of the different restriction enzyme sites and corresponding TALEN pairs. The murine Ugt1 exon 4 and flanking introns is shown in the figure. The relative position of the NlaIV, NcoI, BstXI and MnlI restriction sites is indicated, as well as the left and right arm of each TALEN pair. Exonic and intronic sequences are indicated in yellow and white, respectively.

Therefore, to tackle the problems in setting up the Surveyor assay, we modified the strategy by targeting restriction enzyme (RE) sites present in the exon 4 of the mouse Ugt1 gene. TALEN-induced double strand break will result in the disruption of the RE site by error-prone NHEJ repair. In this case, digestion of the PCR product with the RE will result in a fraction of the PCR product that will be RE-resistant. We designed and constructed four (4) TALEN pairs targeting restriction sites present in the exon 4 of the Ugt1 gene (NlaIV, NcoI, BstXI and MnlI, Figure 1). The selected restriction sites were located in the spacer region, between the two TALEN monomers. The sequences recognized by the different TALENs are indicated in Figure S3. To construct the TALEN plasmids we used the “Golden Gate TALEN and TAL effector Kit 2.0 [11], based on the GoldyTALEN scaffold [12]

### Testing TALEN pairs activity with a luciferase reporter plasmid

**Figure 2:**
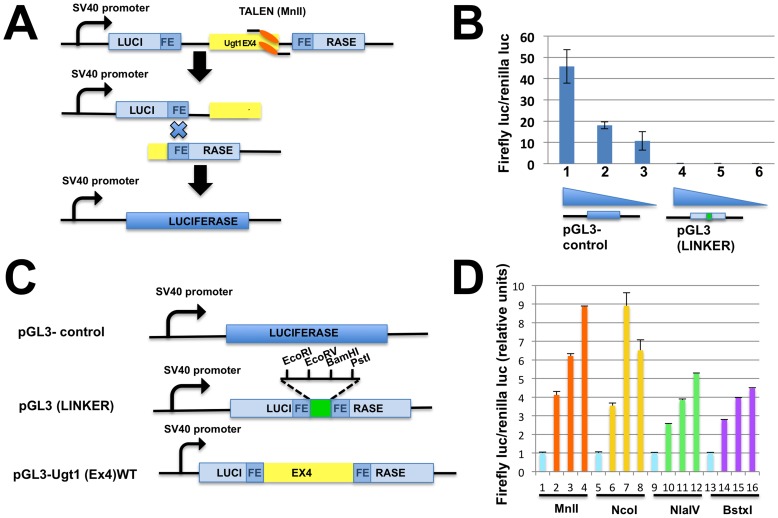
Evaluation of TALEN activity using a Luciferase-reporter plasmid. **A)** Scheme of the TALEN reporter plasmids and strategy. The Ugt1 exon 4 and flanking introns were introduced into the polylinker of the pGL3 (Linker) plasmid. This plasmid contains a 548 bp-repeated region of the luciferase cDNA. DSB induced by TALENs stimulates homologous recombination between the repeated luciferase regions and results in the recovery of luciferase enzyme activity. **B) The reporter plasmid without TALENs showed no luciferase activity.** 0.4 µg, 0.8 µg and 1.6 µg of pGL3-control and pGL3 (Linker) plasmids (lanes 1–3 and 4–6, respectively) were co-transfected with the 40 ng of renilla-luc plasmid into HEK293 cells. The pGL3 (Linker) plasmids showed no luciferase activity (lanes 4–6). **C) Scheme of the reporter plasmid used in the TALEN assays.** The figure shows the schemes of the pGL3-control, pGL3 (Linker) and pGL3-Ugt1(Ex4)WT plasmids. **D) The NcoI TALEN pair shows the highest activity.** The MnlI, NcoI, NlaIV and BstXI TALEN pairs were co-transfected into HEK293 cells together with the reporter pGL3-Ugt1 (Ex4)WT and renilla luc plasmids. Increasing amounts of the TALEN plasmids were co-transfected (200 ng for lanes 2, 6, 10 and 14; 400 ng for lanes 3, 7, 11, and 15; 800 ng for lanes 4, 8, 12, and 16). Activity was normalized to lanes 1, 5, 9, and 13, which contain pGL3 (linker) co-transfected with the higher amount of the respective TALEN pair.

To determine the efficiency of the different pairs of TALENs, we constructed a reporter vector containing a duplicated region of the firefly luciferase cDNA, with the exon 4 of the Ugt1 gene inserted in between the repeated luciferase regions (Figure 2A). Double strand breaks generated by TALENs in the exon 4 will favor the recombination events within the luciferase gene and, in turn, restore the correct primary sequence in the reporter vector. Efficiency of the different TALEN pairs will be then determined as luciferase activity.

We first transfected N-Muli cells with the pGL3-(Linker) reporter vector without insert and verified the absence of luciferase activity (Figure 2B). Then, we co-transfected the reporter vector containing the Ugt1 exon 4 [pGL3-UGT1a1-(Ex4)-WT] together with the different TALEN pairs and determined the activity of the reporter plasmid. Transfection of all four TALEN pairs resulted in sustained luciferase activity, with the NcoI-TALEN being the most active one, followed by the MnlI-TALEN. The NlaIV- and BstXI-TALEN pairs showed lower luciferase activity, that amounted to about half of the activity observed for the MnlI- and NcoI-TALEN pairs (Figures 2C and 2D). The renilla-luciferase vector was used to normalize for differences in transfection efficiency.

### Transient transfection of NcoI TALEN pair results in short deletions at the NcoI site

We disrupted the Ugt1 gene in N-Muli mouse hepatoma cells by transiently transfecting the most effective TALEN pair, the NcoI-TALEN. N-Muli cells were selected because Ugt1 expression levels were high enough to be detected by Western blot (see below). Due to the low transfection efficiency of N-Muli cells (between 15 and 20%, data not shown), we used a vector expressing the GFP protein, together with the TALEN plasmids, to co-transfect the cells. GFP-positive cells were sorted by FACS, genomic DNA was prepared, the exon 4 region of the Ugt1 gene was PCR amplified and the amplicon analyzed by RE digestion.

**Figure 3:**
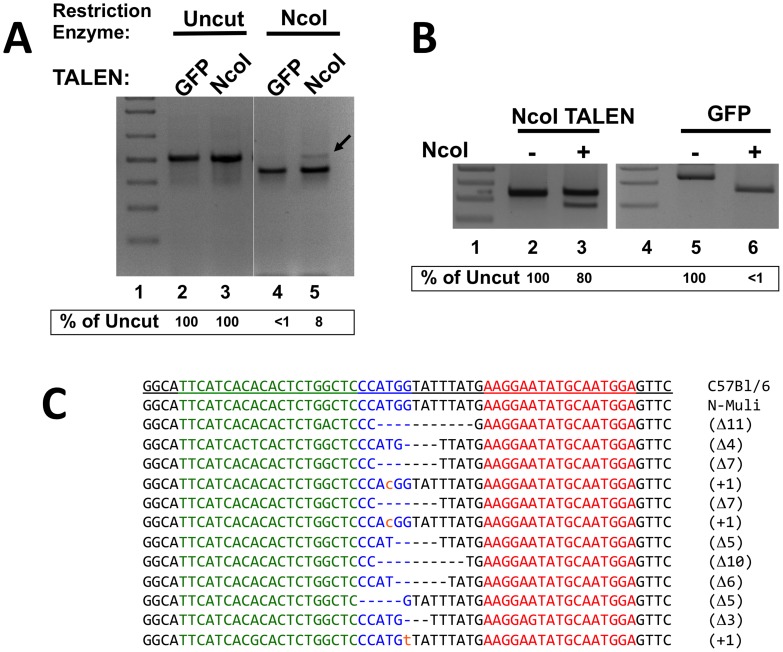
Transient transfection of NcoI TALENs into N-Muli murine hepatoma cells. **A)** Determination of TALEN activity by RE-digestion of genomic PCR product. N-Muli cells were transfected with the NcoI TALEN pair together with a GFP plasmid. GFP positive cells were sorted, genomic DNA prepared and PCR amplified. PCR products were digested or not with NcoI, and the digestion product run in a 2% agarose gel. The arrow indicates the NcoI-resistant fraction of the PCR product to the NcoI-TALEN transfected cells (Lane 5). **B) Confirmation of the presence of NcoI-resistant fragment.** The agarose regions corresponding to the full-length undigested fragment of the gel shown in Panel A (GFP and NcoI-TALEN, lanes 4 and 5, respectively) were cut and DNA purified. A nested PCR reaction using internal primers was performed, and the PCR product was digested or not with NcoI (lanes 3, 6 and 2, 5, respectively). The percentage of uncut fragment is indicated bellow the gel. **C) INDEL analysis of NcoI-TALEN N-Muli treated cells.** The NcoI-resistant fragment of Panel A, Lane 5 was purified, cloned into a pUC19 vector and the obtained clones were sequenced. The left and right arms of the TALEN pair are indicated in green and red, respectively. The NcoI restriction enzyme site is indicated in blue. The deletions are indicated as black dashes. The type and length of the modification are indicated on the right of each sequence (Δ indicates deletion, and + base substitution, indicated by small orange caps).

We observed that the PCR product amplified from GFP-transfected N-Muli cells was completely digested by NcoI while, in the cells transfected with the NcoI TALENs, a fraction of the PCR product was resistant to NcoI digestion (estimated in ∼8%, Figure 3A, lane 5). To further confirm the inactivation of the NcoI site by TALENs, the NcoI-resistant fragment was purified from the agarose gel, PCR amplified with an internal pair of primers (nested PCR), and the PCR product was digested with NcoI. As a negative control, we purified the PCR product originating from cells transfected only with the GFP plasmid. When the PCR products were digested with NcoI, we observed complete digestion of the band derived from the GFP-transfected cells, while 80% of the nested-PCR band originating from the TALEN-transfected cells was resistant to NcoI digestion (Figure 3B), indicating that the band shown in Figure 3A, Lane 5 was resistant to NcoI digestion due to inactivation of the NcoI site. To characterize the INDELs generated in the N-Muli cells, we cloned and sequenced the PCR product of the NcoI resistant fraction (Figure 3A, Lane 5). We observed that the majority of the mutations were deletions of the target region ranging from 3 to 11 bases, with the exception of three clones containing single base mutations (Figure 3C). On the contrary, no insertions were observed. All deletions and mutations were in the spacer region and, as expected, in all cases inactivated the NcoI restriction enzyme site.

### Isolation and screening of Ugt1 deficient clones

To overcome the obstacles caused by the low transfection efficiency of N-Muli cells, we adopted a two-steps strategy based in the co-transfection of the TALEN plasmids with a second vector encoding for a selection marker (see Figure S4 for a flow diagram of the method). First, we co-transfected the cells with the pEGFP-C2 plasmid, which encodes for the Neomycin selection marker, together with the TALEN vectors. G418-resistant cells were selected for 12–13 days (when G418 cells reached about 70% confluence). Second, G418-resistant cells were then transfected with the same TALEN plasmids and a second plasmid encoding for another selection marker (Puromycin gene). Transfected cells were grown in the presence of both antibiotics (G418 and puromycin) and individual clones were isolated and amplified.

**Figure 4:**
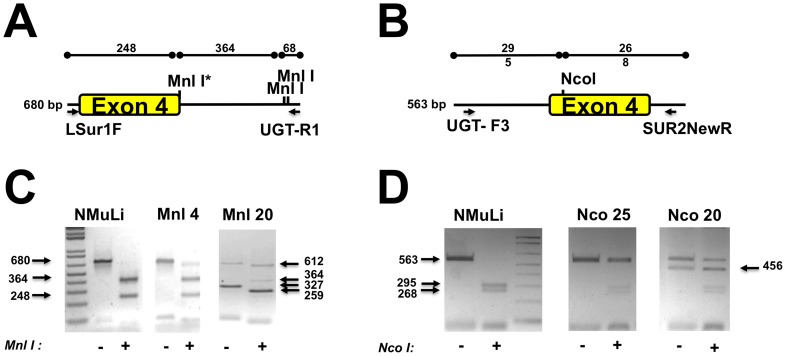
Analysis of N-Muli Ugt1-mutant cell clones. **A) and B)** Schemes of the PCR fragments showing the position of the PCR primers and restriction sites. The position of the MnlI and NcoI sites are indicated (Panels A and B, respectively), as well as the expected length of the digestion products. **C) and D) Restriction enzyme assay to detect INDELs.** DNA from antibiotic-resistant clones (Panels C and D, from MnlI and NcoI TALEN-transfected cells, respectively) was prepared, PCR amplified, digested or not with MnlI and NcoI and the products run in a 1% agarose gel. The expected and obtained gel bands are indicated with arrows. N-Muli corresponds to control DNA, Mnl4, Mnl20, Nco25 and Nco20 to antibiotic-resistant clones that were transfected with the respective TALEN pairs.

We tested the four TALEN pairs mentioned above (NcoI, MnlI, BstXI and NlaIV) in N-Muli cells and analyzed 30, 30, 12 and 10 G418/puromycin resistant clones of each transfection, respectively (Table 1). As an example, the analysis of two NcoI and two MnlI clones are shown in Figure 4. The region of interest was a 680 bp-long fragment and contained one MnlI site located in exon 4 of the Ugt1 gene, which was targeted by the MnlI-TALEN, and two other MnlI sites located in the 3′ flanking intron (Figure 4A). Complete digestion was observed when the amplicon obtained from untransfected N-Muli cells was treated with MnlI, while a fraction of the PCR product from Clone Mnl4 was resistant to MnlI digestion (Figure 4C) suggesting the presence of a mono-allelic mutation. In the case of Clone Mnl20, we obtained a shorter PCR product in addition to the expected PCR product, suggesting the presence of a larger deletion in one of the alleles. Both bands were digested by MnlI at the site present in the intron at the 3′ end of the amplicon, but were not cut at the MnlI target site located at the 5′ splicing site of Exon 4. These results suggest the presence of a large deletion in one of the alleles, and the inactivation of the MnlI site in the other allele.

**Table 1:** Generation of Ugt1 N-Muli mutant cells by TALENs.

TALEN name	Total N. of clones isolated	Total N. of clones analyzed	Total N. of RE-resistant clones	TALEN efficiency (% of mutated cell clones)	TALEN efficiency (obtained with the reporter CFP vector, relative to NlaIV)
NlaIV	10	10	0	0	1.0
NcoI	77	30	11	36.6	2.32
BstXI	12	12	2	16.6	1.03
MnlI	72	30	9	30	1.62

The total number of clones isolated, analyzed and containing the RE site modified are indicated for all TALEN pairs used. The comparison between the TALEN pair efficiency based on the frequency of mutated cell clones obtained and that obtained with the reporter vector is shown.

In the case of the NcoI RE approach, only one NcoI site was present in the 563 bp-long PCR fragment containing Ugt1 exon 4 (Figure 4B). Digestion of the wt fragment resulted in two bands of 295 bp and 268 bp (Figure 4D). Digestion of the PCR product of Clone Nco25A showed the presence of a NcoI-resistant fraction of the amplicon, suggesting the presence of a mono-allelic mutation. Similarly to that observed for the Mnl20 clone, we observed two PCR products for the Nco20 clone. Both bands were resistant to NcoI digestion, suggesting the presence of a large deletion in one of the alleles, and the inactivation of the NcoI site in the other allele. As previously observed in the Mnl20 clone, we observed faint bands corresponding to the digestion products of the wt amplicon.

### Molecular analysis of Ugt1-deficient clones

**Figure 5:**
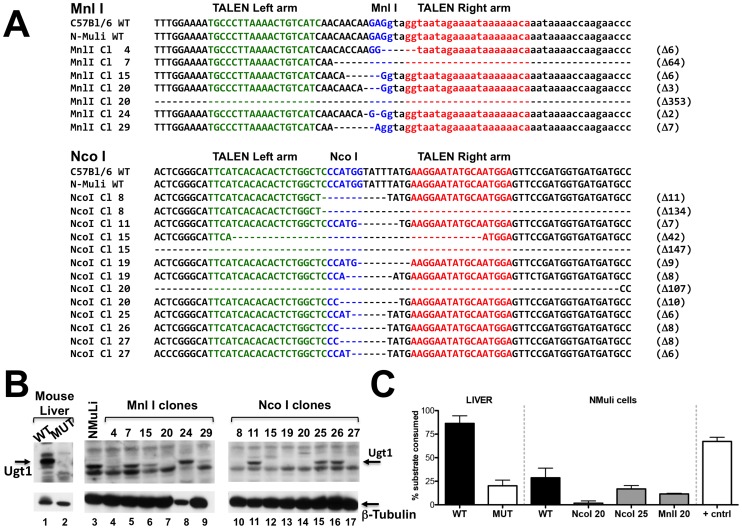
Molecular analysis of cell clones. **A)** INDEL analysis of MnlI-TALEN and NcoI-TALEN N-Muli treated cells. The left and right arms of the TALEN pair are indicated in green and red, respectively. The restriction enzyme sites are indicated in blue. Deletions are indicated as dashes. The length of the deletions is indicated on the right of each sequence. **B) Western blot analysis of the isolated cell clones.** Proteins were prepared from the isolated cell clones and analyzed by Western blot using an anti-Ugt1 antibody. Lanes 1 and 2 correspond to protein extracts prepared from WT and mutant livers [13]. Lane 3 corresponds to untreated N-Muli cells. The arrow indicates the band corresponding to Ugt1. β-tubulin was used to normalize for protein loading. **C) Ugt1 glucuronidation activity of the isolated cell clones.** Ugt1 glucuronidation activity was determined by the UGT-Glo Assay as described in the Materials and Methods section. The activity determination was determined with microsomes prepared from wt and Ugt mutant mouse liver (1 µg), from wt N-Muli cells (10 µg) and from the isolated cell clones (10 µg, Mnl20, Nco20 and Nco25), as the percentage of luminescent substrate consumed. “+cntrl” indicates microsomes provided by the manufacturers (Promega) as positive control of the experiment. The experiment was repeated twice, and the mean of both experiments is shown.

To characterize the genetic defect generated by TALENs we amplified, cloned and sequenced the Ugt1 exon 4 and flanking introns from the isolated cell clones. Both in the MnlI and NcoI clones we observed the presence of deletions, most of them ranging from 2 to 11 bases (Figure 5A). However, we found larger deletions of 353 bp, 64 bp, 42 bp, 147 bp and 107 bp in the Mnl20, Mnl7, Nco15 (both alleles) and Nco20 clones, respectively, confirming the results obtained by PCR of genomic DNA. We found bi-allelic mutations in the Nco8, Nco15, Nco19, Nco20 and Nco27 clones (Figure 5A).

Next, we determined Ugt1 protein levels in the selected cell clones. We prepared protein extracts from the isolated clones and performed a WB analysis to determine the presence of the Ugt1 protein. We observed a significant reduction in protein levels in 3 MnlI clones (Figure 5B, Mnl4, Mnl15 and Mnl29, while no Ugt1 band was detected in the Mnl20 clone. Furthermore, 5 out of 8 NcoI clones analyzed lacked the Ugt1 band (Figure 5B, Nco8, Nco15, Nco19, Nco20 and Nco27). These results confirmed the presence of a bi-allelic mutation in the clones Nco8, Nco15, Nco19, Nco20 and Nco27. The other clones analyzed showed reduced levels of Ugt1 protein compared to the wt protein extract.

We examined Ugt glucuronidation activity using the UGT-Glo Assay, by determining the percent consumption of the UGT multienzyme substrate, as described in “Materials and Methods”. As controls, we used liver microsomes prepared from WT and Ugt1 mutant mice [13]. Mutant mice have no Ugt1 glucuronidation activity due to a targeted mutation in the Ugt1 gene [13]. As expected, substrate consumption was significantly reduced in the Ugt1 KO control (Figure 5C). The residual glucuronidation activity was performed by Ugt2, as the substrate used does not discriminate between Ugt1 and Ugt2 activities. Since N-Muli cells express much lower amounts of Ugt1 than mouse liver (Figure 5B, compare lanes 1 and 3, which correspond to 30 µg and 100 µg of total protein from WT liver and N-Muli cells, respectively), we performed the glucuronidation reaction using a higher amount of microsomes (10 µg from cells and 1 µg from liver). The amount of consumed substrate by N-Muli cells was much lower than in liver microsomes, confirming the lower expression levels of Ugt1 in those cells. We observed that the biallelic mutant clones analyzed (Mnl20 and Nco20) had lower activity than N-Muli cells (Figure 5C). The activity of the monoallelic mutant clone analyzed (Nco 25) was intermediate between WT N-Muli cells and the biallelic clones.

We observed a good correlation between the results obtained with the reporter vector and the number of mutated clones obtained in vivo. In fact, the TALEN-pairs that showed to be highly efficient in the generation of INDELs in vivo, such as those targeting the NcoI and MnlI restriction sites, were also the most efficient ones in re-activating luciferase activity of the reporter vector (Table 1). Finally, the most efficient pair was the NcoI-TALEN, as determined by the NcoI-digestion assay of cell clones (Table 1), by the luciferase activity using the reporter vector (Figure 2D and Table 1), and by the number of clones lacking Ugt1 protein TALENs treatment (Figure 5B and Table 1).

## Discussion

The recent development of highly specific endonucleases opened the possibility to rapidly and efficiently modify the genome of cells, allowing the generation of cell lines having mutations in the gene of interest. These tools are of crucial importance to more deeply understand the mechanisms of disease and the metabolic pathways affected when specific genes are mutated. In the present work we generated specific TALEN pairs and tested their endonuclease efficiency in N-Muli murine liver-derived tissue culture cells, with the final outcome of creating mono- and bi-allelic mutations of the Ugt1 gene. The rationale of using murine N-Muli cells instead of human cells to produce mutant cell lines resides in the future objective of performing in vivo editing of a Crigler-Najjar mouse model [13]. This mouse strain, which contains a one-base deletion in the exon 4 of the Ugt1 gene resulting in a frameshift and premature stop codon, represents and excellent model to set up gene-editing using TALEN technology.

We report a fast and efficient method to perform gene knockout in tissue culture cells, based on the use of TALEN pairs targeting restriction enzyme sites in the region of interest, as already performed in other systems [18]–[20]. The strategy of using restriction enzyme sites was necessary due to the presence of strain-specific allelic variants and pseudogenes [15], [17], a condition that impeded the use of the Surveyor assay to detect INDELs. In addition, we presented a luciferase vector designed to rapidly and accurately determine the efficiency of the generated TALEN.

The efficiency of generating double strand breaks and INDELs by ZNF, TALEN and CRISPR/CAS9 systems is usually determined by the mismatch assay (also called Surveyor, CEL-1, or Nuclease S assays), a method based in the detection and digestion by an endonuclease of mismatches in heterodimers formed between PCR-amplified DNA fragments from control and treated samples [21]. However, performing this technique in animal models faces the problem of the presence of strain-specific polymorphisms. Most targeted mutations are performed in 129/Sv-derived ES cells, but mutated alleles are then transferred by successive backcrossing into a more widely used strain such as C57Bl/6, which is one of the best characterized inbred strains and the reference strain for the mouse genome sequence database [22]–[24]. Consequently, the wt gene of interest originates from a different genetic background compared to the mutant one. Thus, strain-specific allele polymorphisms may limit the use of the Surveyor assay to determine the efficiency of the gene editing/gene KO, if strain-specific alleles are present. In addition, the presence of pseudogenes may complicate the analysis even more. We have shown here that these issues could be avoided by targeting restriction enzyme sites in the region of interest. To disrupt the Ugt1 gene in N-Muli cells, we constructed TALEN pairs targeting four different restriction sites present in Exon 4. Despite the use of the same parameters to design the different TALEN pairs, our luciferase reporter assay showed that the cutting efficiency among enzymes varied substantially, suggesting the need of testing diverse TALEN-pair combinations in order to find the most efficient one. The causes for the variability are not clear, but may be related to the array length and composition [11]. Though, the two TALEN pairs that most efficiently rescued Luciferase activity of the reporter vector (MnlI and NcoI TALEN pairs) were also the most efficient ones in generating mutations in the Ugt1 exon 4 in the genome context of N-Muli cells, obtaining a success rate of about 30% and 37% of mutated clones with those enzymes, respectively. Therefore, we reported a very good correlation between luciferase activity of the reporter vector and the number of clones having mutations in the exon 4 of the Ugt1 gene for the four TALEN pairs tested. Several cell clones with bi-allelic modification of the Ugt1 gene were devoid of Ugt1-specific band in the Western blot analysis, confirming the drastic nature of the generated mutations. In the case of the MnlI site, it coincides with the 5′ splicing site of Ugt1 exon 4. Consequently, it is expected that mutations in this site may affect pre-mRNA processing [25]. However, semi-quantitative RT-PCR failed to detect important differences in Ugt1 mRNA levels (data not shown). More experiments should be performed to determine whether cryptic sites nearby are activated. In the case of the NcoI site, most of the analyzed mutations disrupted the natural reading frame, confirming the WB results where most clones contained bi-allelic mutations. Truncated proteins lacking the C-terminal portion lose the transmembrane-docking domain, resulting in an inactive protein [4], [13], [26]–[28]. In addition, despite of the fact that the TALEN-encoding plasmids did not confer any selective advantage to the cells (the selection markers were encoded by different plasmids), we cannot not exclude the eventual integration of the TALEN-encoding plasmids in the genome and further experiments should be performed to rule out this possibility.

The study of glucuronosyl transferase enzyme activity was originally performed in microsomes from patients' liver [4], [28], [29] and is now normally carried out in tissue culture cell lines, such as COS-1, COS-7, fibroblasts or HEK293 cell lines, stably or transiently transfected with plasmids expressing the specific cDNAs [28], [30]–[33]. These cell lines are used to study substrate specificity [32], the requirement of specific co-factors and the potency of inhibitors [34], and to correlate enzyme activity to the presence of specific mutations in the gene [33]. However, one limitation of this approach is that they do not generally derive from the original tissue expressing the enzymatic activity. In that respect, the cell lines generated in the present work lack Ugt1 glucuronidation activity and could be a very useful tool to perform the studies mentioned above and other pharmacological aspects in a more natural context, by adding back to the mutant cell clones specific variants of the Ugt1 gene.

To summarize, the mutant liver-derived cell lines generated in the present work represent a step forward to study biochemical aspects of Ugt1 enzyme activity in a more natural context. We were able to generate efficient TALEN pairs and to determine the most efficient TALEN pair despite of the presence of strain-specific polymorphisms and pseudogenes. This step, needed to target the Ugt1 gene, is a fundamental one towards setting up the gene editing technology, in order to find alternative therapies to treat the Crigler-Najjar syndrome type I.

## Supporting Information

Figure S1 Surveyor analysis of plasmid and genomic DNA sequences. PCR products of WT and MUT plasmids, which contain the murine Ugt1 Exon 4 and flanking sequences differing by one nucleotide [13], were treated or not with Surveyor (lanes 2–3 and 12–13, respectively). Cleavage of the heterodimers (Lane 4) resulted in the expected DNA fragments of 192 and 324 bp. PCR products from WT and Ugt1-MUT mice were mixed and treated or not with Surveyor (Lanes 7 and 11, respectively).
(TIF)

Figure S2 Sequence of the WT exon 4 and mutation analysis by Surveyor. Alignment of reference GenBank sequence (C57Bl/6 strain) of WT exon 4 (capital letters) and flanking intron sequences (lower case) with that of 129/SvJ clones. DNA from 129/SvJ WT ES cells was PCR amplified, the PCR product cloned into pUC19 and sequenced. The differences between the GeneBank and 129/SvJ sequence are indicated with the red boxes. The SNPs present in the region are indicated with the light blue boxes. The base deleted in mutant mice is marked by a blue rectangle. The sequences were grouped according to the variations present (indicated by the blue triangles and red stars, in the left).
(TIF)

Figure S3 DNA sequence of the murine exon 4 and flanking introns. The sequence of the murine Ugt1 exon 4 and flanking introns is represented (capital letters and small caps, respectively). The exon is indicated in red. The restriction sites of NlaIV, NcoI, BstXI and MnlI, and position of the left and right arms of the different TALEN pairs are indicated. The primers used for the genomic PCR are indicated with arrows, and the sequence underlined.
(TIF)

Figure S4 Scheme of the strategy used to obtain Ugt1-mutant cell clones. The TALEN pair for each RE site was co-transfected into N-Muli cells together with a plasmid encoding for the Neomycin-resistance gene. G418-resistant cells (mixed population) were grown and co-transfected with the same TALEN pair and a second plasmid with a Puromycin-resistance gene. Individual cell clones resistant to both antibiotics were isolated and analyzed. The MnlI TALEN pair is shown as example.
(TIF)

Table S1 List of primers used in the genome PCR of the isolated cell clones.
(DOCX)
