# Supplementary figures and images for: Generation of Ugt1-Deficient Murine Liver Cell Lines Using TALEN Technology
Source: PLoS One. 2014 Aug 13;9(8):e104816. doi: 10.1371/journal.pone.0104816 (PMC4132024; doi:10.1371/journal.pone.0104816)

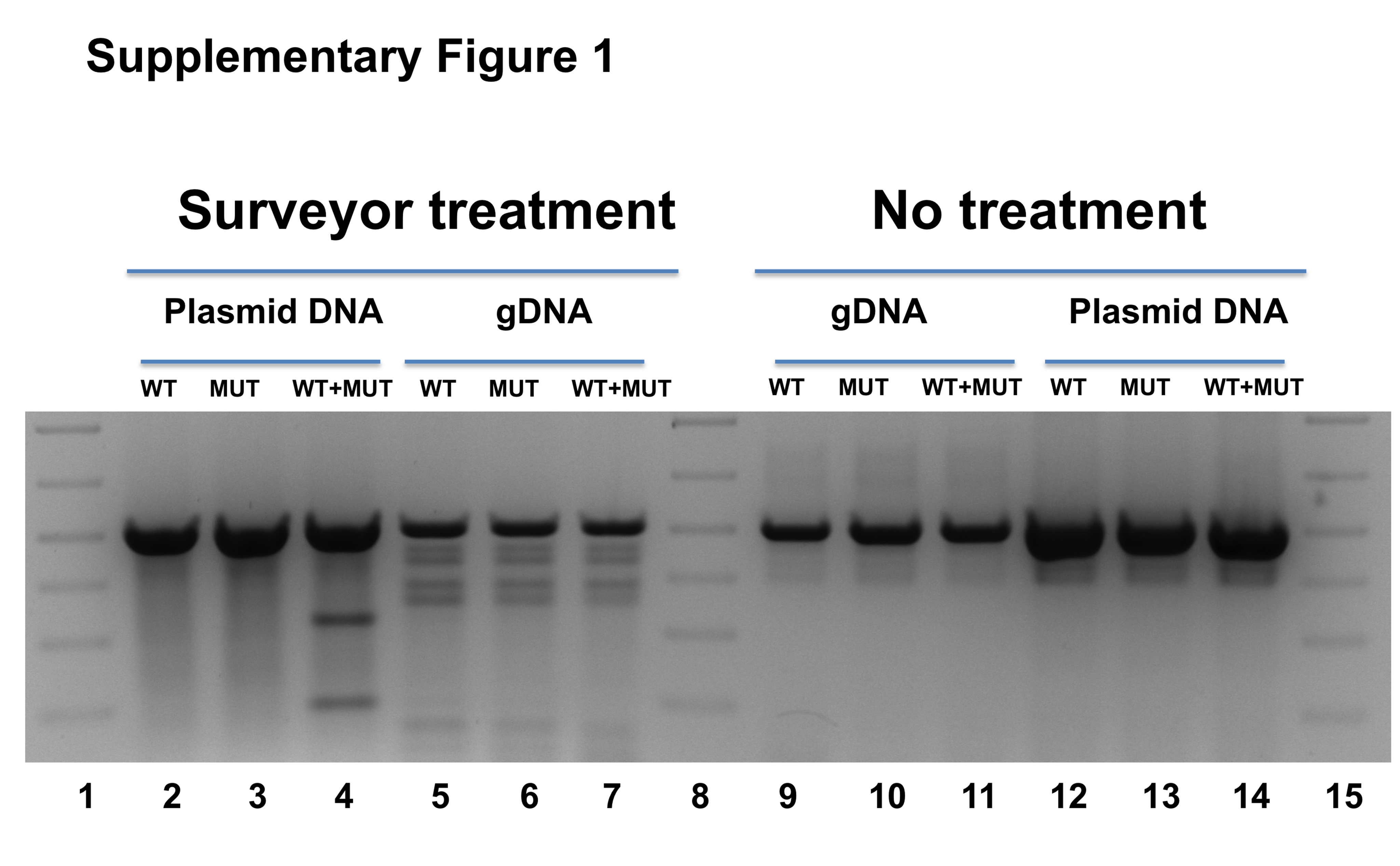

Supplement: Figure S1 — Surveyor analysis of plasmid and genomic DNA sequences. PCR products of WT and MUT plasmids, which contain the murine Ugt1 Exon 4 and flanking sequences differing by one nucleotide [13], were treated or not with Surveyor (lanes 2–3 and 12–13, respectively). Cleavage of the heterodimers (Lane 4) resulted in the expected DNA fragments of 192 and 324 bp. PCR products from WT and Ugt1-MUT mice were mixed and treated or not with Surveyor (Lanes 7 and 11, respectively). (TIF) [file pone.0104816.s001.tif]

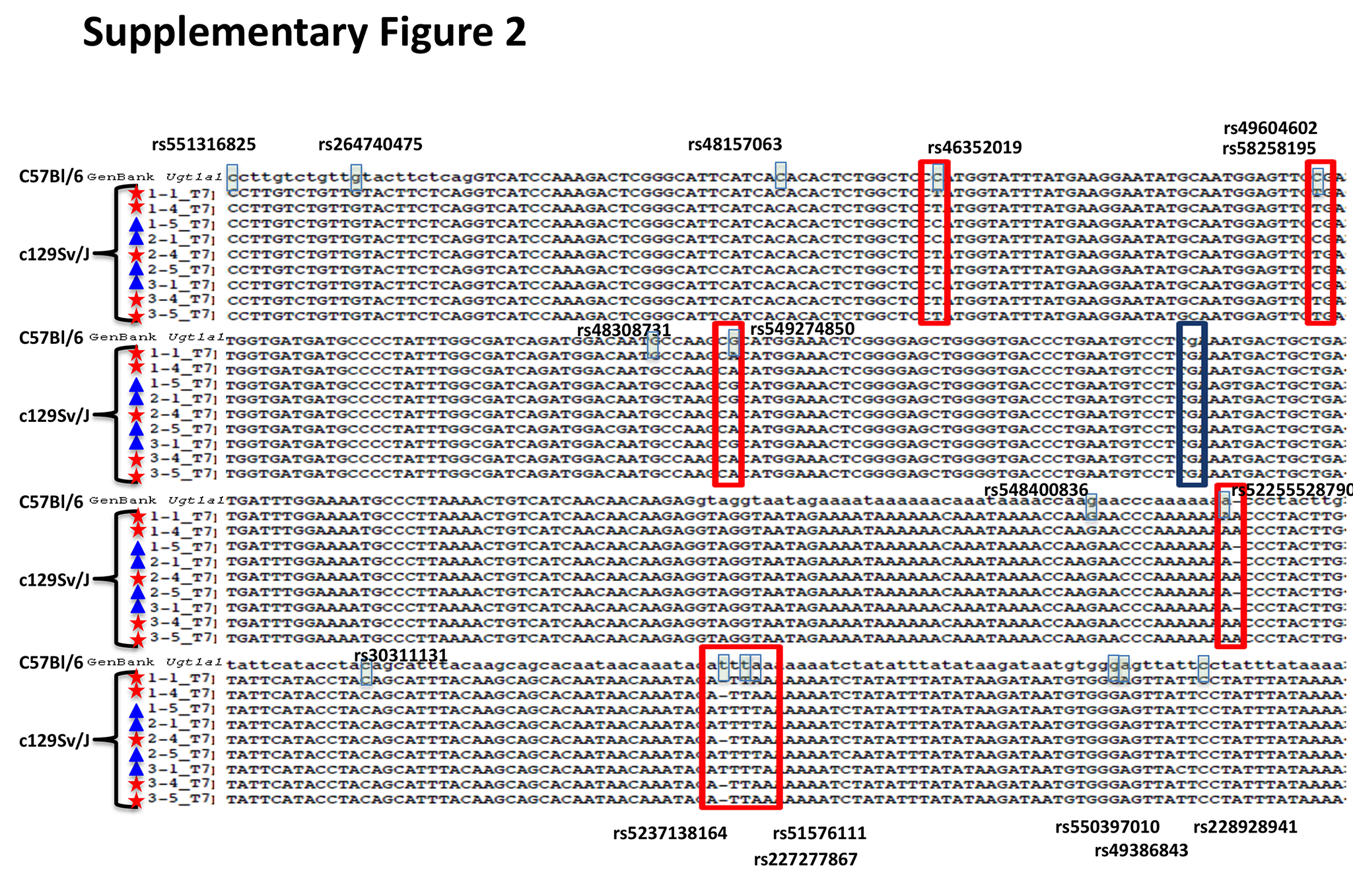

Supplement: Figure S2 — Sequence of the WT exon 4 and mutation analysis by Surveyor. Alignment of reference GenBank sequence (C57Bl/6 strain) of WT exon 4 (capital letters) and flanking intron sequences (lower case) with that of 129/SvJ clones. DNA from 129/SvJ WT ES cells was PCR amplified, the PCR product cloned into pUC19 and sequenced. The differences between the GeneBank and 129/SvJ sequence are indicated with the red boxes. The SNPs present in the region are indicated with the light blue boxes. The base deleted in mutant mice is marked by a blue rectangle. The sequences were grouped according to the variations present (indicated by the blue triangles and red stars, in the left). (TIF) [file pone.0104816.s002.tif]

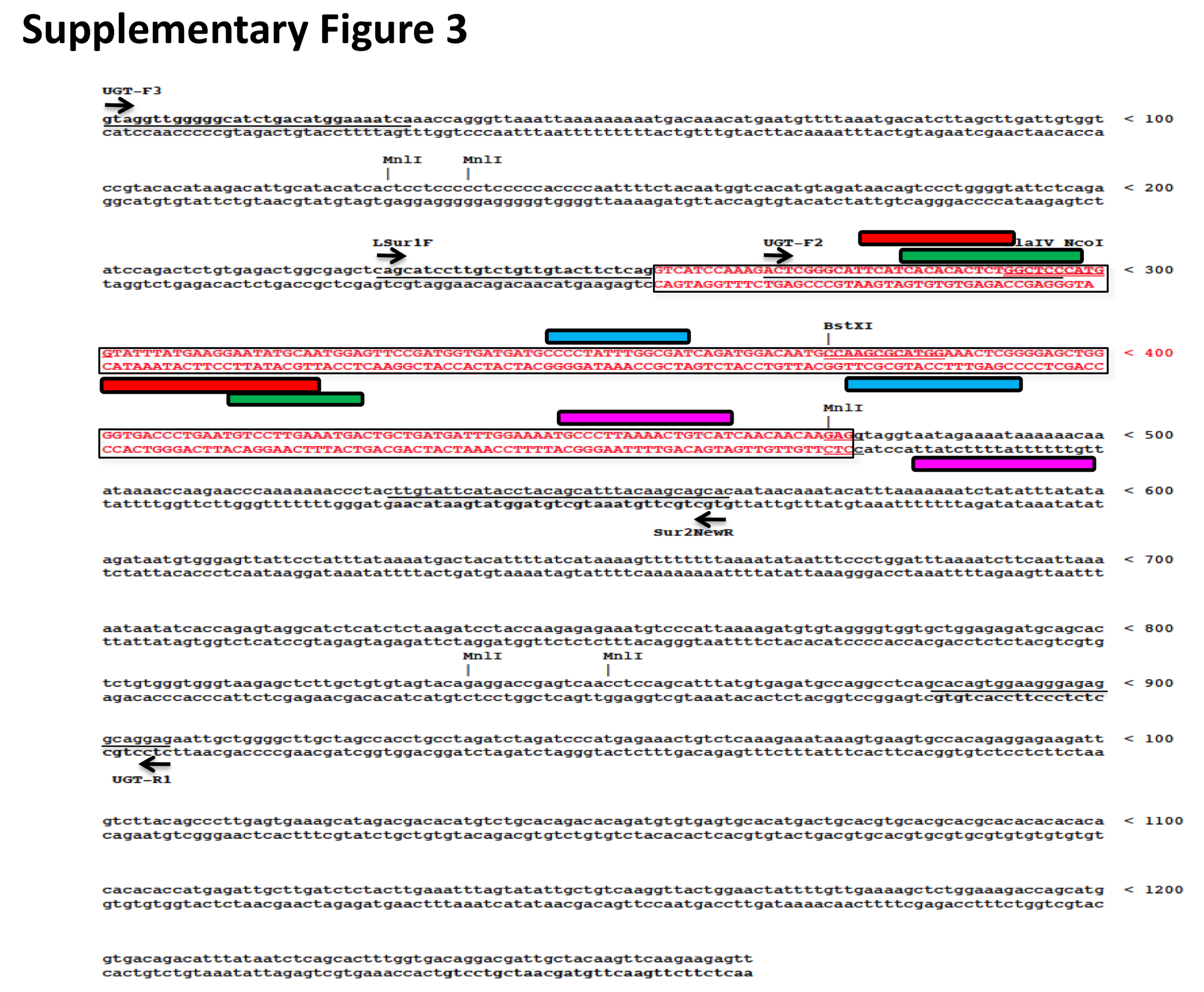

Supplement: Figure S3 — DNA sequence of the murine exon 4 and flanking introns. The sequence of the murine Ugt1 exon 4 and flanking introns is represented (capital letters and small caps, respectively). The exon is indicated in red. The restriction sites of NlaIV, NcoI, BstXI and MnlI, and position of the left and right arms of the different TALEN pairs are indicated. The primers used for the genomic PCR are indicated with arrows, and the sequence underlined. (TIF) [file pone.0104816.s003.tif]

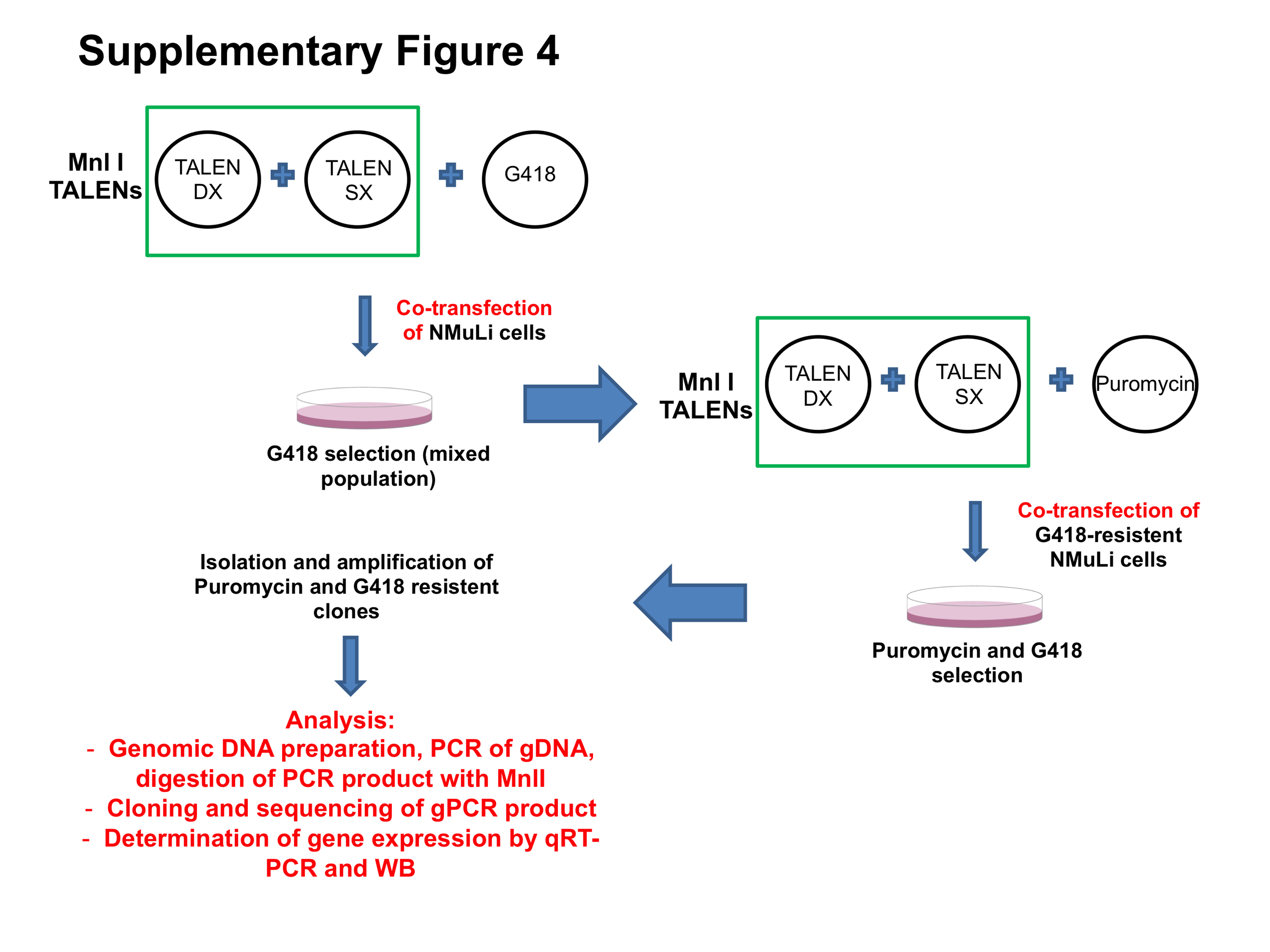

Supplement: Figure S4 — Scheme of the strategy used to obtain Ugt1-mutant cell clones. The TALEN pair for each RE site was co-transfected into N-Muli cells together with a plasmid encoding for the Neomycin-resistance gene. G418-resistant cells (mixed population) were grown and co-transfected with the same TALEN pair and a second plasmid with a Puromycin-resistance gene. Individual cell clones resistant to both antibiotics were isolated and analyzed. The MnlI TALEN pair is shown as example. (TIF) [file pone.0104816.s004.tif]
