# Supplementary material for: Generation of Ugt1-Deficient Murine Liver Cell Lines Using TALEN Technology
Source: PLoS One. 2014 Aug 13;9(8):e104816. doi: 10.1371/journal.pone.0104816 (PMC4132024; doi:10.1371/journal.pone.0104816)
Supplement: Table S1 — List of primers used in the genome PCR of the isolated cell clones. (DOCX) [file pone.0104816.s005.docx]

**Supplementary Table 1**

| **Primer** | **Sequence (5’-3’)** | **Length (bp)** |
| --- | --- | --- |
| **LSur1R** | GGT AGG ATC TTA GAG ATG AGA TGC CTA CTC TGG TG | 35 |
| **LSur1F** | CAG CAT CCT TGT CTG TTG TAC TTC TCA GGT CAT CC | 35 |
| **Sur2NewR** | GTG CTG CTT GTA AAT GCT GTA GGT ATG AAT ACA AG | 35 |
| **UGT F1** | GAG TTC CGA TGG TGA TGA TGC CCC TAT TTG | 30 |
| **UGT F2** | ACT CGG GCA TTC ATC ACA CAC TCT GG | 26 |
| **UGT F3** | GTA GGT TGG GGG CAT CTG ACA TGG AAA ATC A | 31 |
| **UGT R1** | CTC CTG CCT CTC CCT TCC ACT GTG C | 25 |
| **UGT R2** | ACT CTT CTT GAA CTT GTA GCA ATC GTC CTG | 30 |

**Supplementary Table 1.** List of primers used in the genome PCR of the isolated cell clones.
